# The impact of regulatory T cells on carcinogen-induced sarcogenesis

**DOI:** 10.1038/sj.bjc.6603824

**Published:** 2007-06-12

**Authors:** G Betts, J Twohig, M Van den Broek, S Sierro, A Godkin, A Gallimore

**Affiliations:** 1 Medical Biochemistry and Immunology, Henry Wellcome Building, Heath Park, Cardiff, CF14 4XN, UK; 2 Institute of Experimental Immunology, Schmelzbergstrasse 12, CH-8091, Zurich, Switzerland; 3 Peter Medawar Building for Pathogen Research, South Parks Road, Oxford, OX1 3SY, UK

**Keywords:** immune surveillance, regulatory T cells, methylcholanthrene

## Abstract

Burnet proposed in the 1950's that the immune system is engaged in identifying and destroying abnormal cancerous cells. This process, termed immune surveillance, has been at the centre of intense debate for decades. Results using immunodeficient mice lend support to the immune surveillance hypothesis. We surmised that immune surveillance would be hampered by the inhibitory effect of naturally occurring FoxP3^+^ regulatory T cells, a population of T cells shown to be present at an increased frequency in a variety of human tumours. The carcinogen, methylcholanthrene was injected subcutaneously into mice and the steady development of fibrosarcomas was observed over approximately 200 days. These fibrosarcomas were strikingly infiltrated with FoxP3^+^ regulatory T cells implying that these cells impinge upon immune-mediated rejection of the tumour. This was confirmed by partial ablation of FoxP3^+^ regulatory T-cell activity, which resulted in a marked reduction in tumour incidence. The reduction of tumour incidence was ablated in mice that lacked interferon gamma. These data offer strong support for the concept of immune surveillance and indicate that this process is limited by the inhibitory effect of FoxP3^+^ regulatory T cells.

Burnet hypothesised in the 1950's that the adaptive immune system could control and eliminate developing tumours, a process later termed tumour immune surveillance (Burnet, 1957). Although this theory subsequently fell from popularity, a large body of recent work from different groups has demonstrated an increase in both spontaneous and carcinogen-induced tumours in immunocompromised mice including those lacking T cells, natural killer cells (NK) and natural killer T cells (NKT), type 1 and type 2 interferons, perforin and the IFN*γ* receptor (IFN*γ*R); (reviewed by Smyth *et al*, 2006). These experiments have largely been interpreted as evidence to support the tumour immune surveillance concept and demonstrate the many arms of the immune system involved in tumour destruction.

CD4^+^ FOXP3^+^CD25^+^ regulatory T cells (Tregs) comprise approximately 10% of the CD4^+^ T-cell population in mice. In humans, the regulatory CD4^+^ T-cell pool is restricted to 1–2% of the CD4^+^ population that are described as CD25^hi^ (Baecher-Allan *et al*, 2001). These cells are thought to be essential for maintaining tolerance to self-antigens recognised by autoreactive T cells that escape deletion in the thymus (Sakaguchi, 2005). An absence of functional Tregs has been associated with several autoimmune diseases in both animal models (Brunkow *et al*, 2001) and humans (Bennett *et al*, 2001). It was hypothesised that the same Tregs would have a derogatory impact on antitumour immunity by inhibiting the activity of T cells that recognise tumour antigens (Shimizu *et al*, 1999). Indeed, several groups, including our own, have shown that depletion of Tregs promotes rejection of tumour cells lines injected into mice (Onizuka *et al*, 1999; Shimizu *et al* 1999; Sutmuller *et al*, 2001; Golgher *et al*, 2002). In addition, evidence from a number of laboratories indicates that higher frequencies of Tregs are observed in the blood of patients with cancer compared to healthy subjects (reviewed in Betts *et al*, 2006). Results of a study of patients with ovarian cancer indicated that the frequency of tumour infiltrating Tregs correlated with the extent of disease thereby implying that Tregs are involved in the pathogenesis of cancer (Curiel *et al*, 2004; Wolf *et al*, 2005). Furthermore, we have also recently found that Tregs appear to control antitumour immune responses in patients with colorectal cancer (Clarke *et al*, 2006). Despite these observational studies in humans, it is still unclear whether the frequency and activity of Tregs increases in response to the tumour or whether tumours are more likely to develop in individuals with higher frequencies of Tregs, thereby raising the question of whether Tregs play a role in tumour immune surveillance. This question cannot be addressed by inoculation of tumour cell lines as these cells are already endowed with the necessary mutational and epigenetic changes required to rapidly produce palpable tumours. Use of these tumours does not allow for significant interactions between the immune system and cells in the process of transformation. Models that use carcinogens do include these early interactions and therefore may be considered more relevant for deconstructing the relationship between the immune system and developing tumours. In addition, the immune system is thought to not only influence tumour outgrowth at the early stages of tumour development but also, through a sustained interaction with the growing tumour, to continuously modify and diminish the immunogenicity of the tumour (Shankaran *et al*, 2001). Thus, cell lines subjected to prolonged periods of *in vitro* culture are almost certainly more immunogenic than tumour cells *in vivo*. With this in mind, it is reasonable to hypothesise that the impact of Tregs on the immune response to tumours developing *in vivo* may differ to their impact on the immune response to tumour cell lines. Injection of the carcinogen methylcholanthrene (MCA) is an established tumour induction model that has been used to examine the role of a series of cell types and signalling molecules that suppress tumour development. Thus, using MCA, we have examined how altered frequencies of Tregs impinge on the development of *de novo* tumours. Specifically, we determined whether (1) Tregs are present in MCA-induced tumours, (2) Tregs influence *de novo* tumour development, (3) IFN*γ* is required for control of tumour growth in Treg depleted mice and (4) depletion of Tregs promotes autoimmunity in MCA-treated mice. The implications of our findings are discussed in the context of tumour immune surveillance.

## MATERIALS AND METHODS

### Mice

Six- to twelve-week-old female wild-type (WT) and IFN*γ*-deficient (IFN*γ*^−/−^) mice on a C57BL/6 (B6, H-2^b^) background were sourced on site. IFN*γ*^−/−^ mice were originally purchased from the Jackson Laboratory (Bar harbour, ME , USA) and kindly donated by Professor N. Topley. Mice were housed in filter top cages and supplied with sterile food and bedding. Experiments were performed in compliance with UK Home Office regulations.

### Methylcholanthrene injections

A total of 400 *μ*g MCA (Sigma-Aldrich, Gillingham, Dorset, UK) suspended in 100 μl olive oil was injected subcutaneously (s.c.) into the hind leg of the mice. Mice were monitored weekly for tumour development.

### Depletion of CD25^+^ cells

Mice were injected intraperitoneally (i.p.) with 0.5 mg of the CD25-specific monoclonal antibody (mAb), PC61 (Lowenthal *et al*, 1985) or the isotype control mAb, GL113 at the indicated time points in 100 μl PBS before MCA administration.

### Flow cytometry

Single-cell suspensions were prepared by filtering mashed tissues through 70 *μ*m nylon strainers (BD falcon, Franklin Lakes, NJ, USA). Cells were stained with anti-CD4-fluorescein isothiocyanate (FITC), anti-CD4-PE Alexa 610, anti-CD25-biotin (7D4)/SA-PerCP-Cy5.5, anti-TCR*αβ* Allophycocyanin (APC), anti-Fc*γ*III/II receptor (BD Franklin Lakes, NJ, USA) and anti-FOXP3-R-Phycoerythrin (PE) using the ebioscience staining kit (ebioscience, San Diego, CA, USA, staining kit) mAbs.

### Suppression assay

CD4^+^ T cells were purified from single-cell suspensions of splenocytes by magnetic associated cell sorting (MACS) (Miltenyi Biotec, Auburn, CA, USA). CD4^+^CD25^+^ cells were separated from CD4^+^CD25^−^ cells by MACS. CD4^+^CD25^−^ cells (2 × 10^4^) were stimulated with 1 *μ*m anti-CD3 mAb (Leinco, St Louis, MO, USA) and 1 × 10^5^ mitomycin (Sigma-Aldrich, Gillingham, Dorset, UK) treated CD4^−^ splenocytes and incubated with titrated numbers of CD4^+^CD25^+^ cells.

### Immunohistochemistry

Cryostat-frozen sections (10 *μ*m) were dried and fixed with cold acetone for 15 min on ice. Sections were permeabilised with 0.2% Tween 20, 1% BSA for 30 min before a 1-h incubation with rabbit anti-mouse foxp3 polyclonal antibodies (Ab) (Roncador *et al*, 2005) and rat anti-mouse mAb (L3T4, BD San Jose, CA, USA) or rat anti-mouse CD45R/B220 mAb (RA3-6B2, BD Pharmingen San Jose, CA, USA) or rat anti-mouse CD8 mAb (53-6.7, BD Pharmingen, San Jose, CA, USA). Sections were subsequently incubated with biotinylated swine anti-rabbit polyclonal Ab (DakoCytomation, Carpinteria, CA, USA) followed by Alexa594-conjugated streptavidin and Alexa488-conjugated goat anti-rat IgG (Invitrogen, Carlsbad, CA, USA). Sections were mounted (Vecta Shield, Burlingame, CA, USA) and examined with using a DM LB2 microscope (Leica, Hicksville, NY, USA) and analysed using the Openlab software package (Improvision, Coventry, Warwickshire, UK).

### Autoantibody detection

Serum was collected from mice and analysed for circulating anti-double-stranded DNA (dsDNA) IgM autoantibody according to the manufacturers’ protocol (Alpha Diagnostic, San Antonio, TX, USA).

### Statistical analysis

The extent of Treg infiltration in different organs was compared using a paired *t*-test. Comparisons of tumour induction between experimental groups were made using a log-ranked test.

## RESULTS

### Treg infiltration of MCA-induced tumours

**Figure 1:**
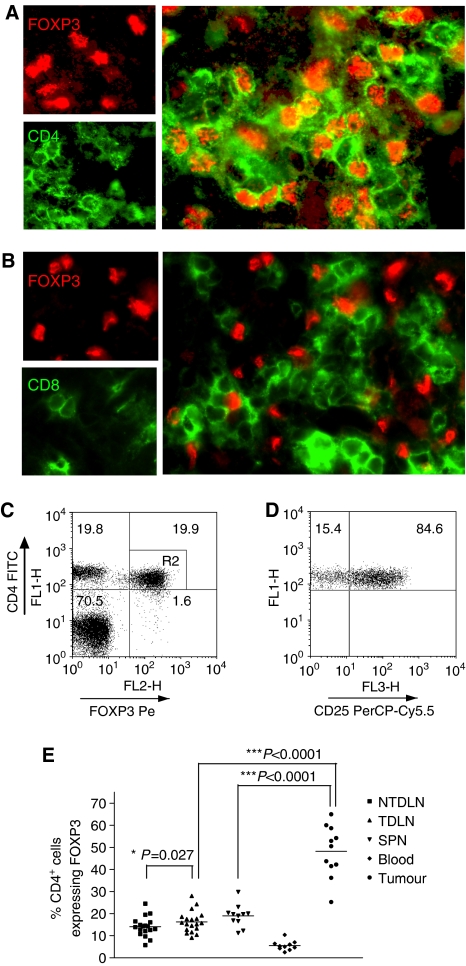
Methylcholanthrene-induced tumours exhibit a significant enrichment of CD4^+^FOXP3^+^ Tregs. Sections of MCA-induced tumours were stained with anti-FOXP3-specific Ab and either anti-CD4 (**A**) or –CD8 (**B**) specific mAb. Single-cell suspensions of TILs, NTDLN, TDLN, spleen and blood were stained with anti-CD4-, anti-CD25- and anti-FOXP3-specific mAbs and analysed by flow cytometry. A representative FACS plot of TIL staining is shown and CD4^+^FOXP3^+^ cells were gated (R2) (**C**). This gate was used to examine CD25 expression as shown in the right panel (**D**). The percentage of CD4^+^ cells expressing FOXP3 in each compartment is presented (**E**). Data were analysed using a paired student's *t*-test.

In preliminary experiments, we found approximately 70–80% of mice develop fibrosarcomas by day 200 after injection of a high dose (400 *μ*g) of MCA. We first sought to determine whether Treg infiltrate MCA-induced tumours. Methylcholanthrene-induced tumours were removed from mice and examined histologically for Tregs by staining sections with Abs specific for FOXP3 and CD4. As shown on Figure 1A, tumours were indeed infiltrated with CD4^+^FOXP3^+^ Tregs, which were in close proximity to CD4^+^FOXP3^−^ conventional T cells (Figure 1A) and CD8^+^ T cells (Figure 1B). The majority of infiltrating lymphocytes were observed at the tumour margin in the interface with healthy leg muscle (data not shown). These data suggest an ongoing immune response to the developing tumour, which may be controlled, to the detriment of the host, by Tregs.

To evaluate further the proportion of Tregs within the tumour infiltrating lymphocytes (TILs), TILs were stained with CD4-, CD25- and FOXP3-specific Abs and analysed by flow cytometry. Supporting the histological observations, approximately half of the CD4^+^ T cells within the TIL expressed FOXP3 (Figure 1C). Approximately 85% of these cells expressed CD25 (Figure 1D), a finding that is in agreement with a previous study, which indicated that, depending on anatomical location, between 20 and 40% of CD4^+^FOXP3^+^ cells, do not express CD25 (Fontenot *et al*, 2005). Comparisons were made of the Treg frequencies in spleen, blood, pooled tumour-draining lymph nodes (TDLN) and non-TDLNs (NTDLN). These data established that Tregs were markedly enriched in TIL compared to the lymphoid tissues, suggesting that they are exerting local control of antitumour immune responses (Figure 1E). A significantly larger proportion of Tregs was also found within the CD4^+^ T cells from pooled TDLN mice compared to pooled NTDLN (Figure 1E). Collectively, these data clearly indicate that Tregs expand in response to the developing tumour and are, as described previously in studies using tumour cell lines (Ghiringhelli *et al*, 2005), enriched in areas of tumour-specific immune activity.

### Influence of Tregs on development of MCA-induced tumours

The markedly high infiltrate of Tregs within the fibrosarcomas led us to explore whether depletion of the Tregs would protect against the development of MCA-induced tumours.

#### Depletion of Tregs using the mAb PC61

**Figure 2:**
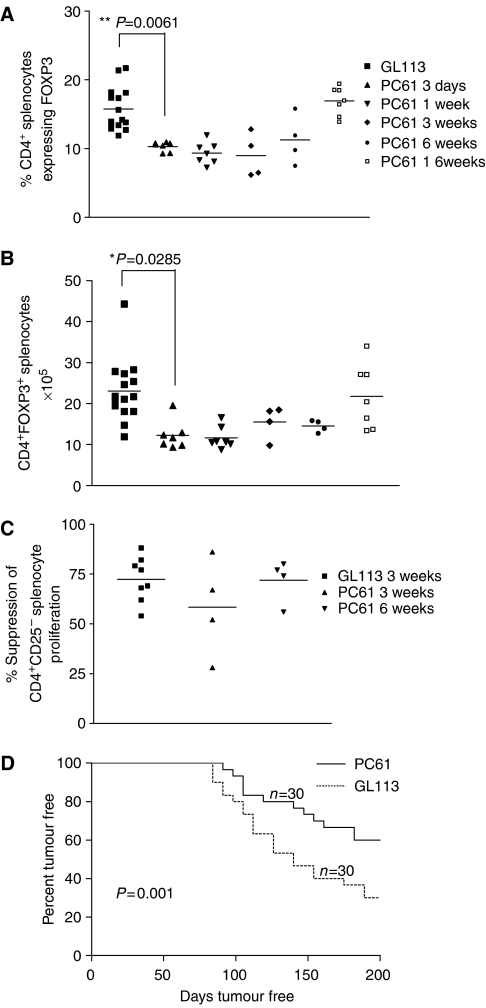
Incidence of MCA-induced tumours is significantly reduced in Treg-depleted mice. The ability of anti-CD25-specific Ab PC61 to deplete CD4^+^FOXP3^+^ Tregs was assessed by analysing the Treg infiltration of spleens following treatment with the mAb PC61 or the isotype control, GL113. Spleens were harvested at 3 days, 1 week, 3 weeks, 6 weeks and 16 weeks after injection and the percentage of CD4^+^ cells that express FOXP3 (**A**), and the total number of CD4^+^FOXP3^+^ splenocytes (**B**) were measured. The suppressive capacity of Tregs following PC61 injection was measured in an *in vitro* suppression assay (**C**). Data were analysed using an unpaired student's *t*-test. Mice were treated with 1 mg of either PC61 or isotype control GL113 mAb before the injection of MCA. The rate of tumour induction, representing the cumulative data from three experiments, is presented (**D**). Log-rank statistical analysis was used to determine the difference in tumour induction between experimental groups.

Depletion of Tregs was carried out using the CD25-specific, depleting mAb, PC61. Phenotypic analyses of PC61-treated mice revealed that administration of PC61 significantly reduced the number and percentage of CD4^+^FOXP3^+^ Tregs in spleens of treated mice (Figure 2A, B). Thus, treatment with PC61 does not completely remove Tregs for, as we outlined above, a proportion of CD4^+^FOXP3^+^ cells express low or no cell surface CD25 (Fontenot *et al*, 2005). CD4^+^CD25^+^ T cells purified from spleens 3 and 6 weeks following administration of PC61 were purified and found to exhibit, on a per cell basis, suppressive capacity comparable to that observed in mice receiving the control mAb, GL113 (Figure 2C).

#### Tumour incidence in PC61-treated mice

Mice treated as described above with either PC61 or control mAbs, were injected 1 day later with MCA mixed in olive oil. Tumour growth was monitored over 200 days. Figure 2D shows pooled data collected from three separate experiments with similar results, including one experiment performed in an animal house at a separate institution. Twice as many mice in the PC61-treated group remained tumour free (18/30) compared to the group treated with control Abs (9/30). These data indicate that even a partial reduction in Treg numbers results in a highly significant reduction in overall tumour incidence and delayed tumour development compared to mice receiving the control mAb, GL113 (*P*=0.001) (Figure 2D). Next, we determined whether additional injections of PC61 would further reduce MCA-induced tumour incidence. Mice were injected with PC61 before MCA administration as described above. Some mice subsequently received further doses of PC61 or isotype control mAb on days 22/24 and 41/43 post-MCA administration. No difference in tumour incidence was observed between the two groups (data not shown) indicating that further injections did not increase the incidence of tumour-free mice. The effect was not due to neutralisation of the rat mAb *in vivo*, as we observed that whereas mouse-anti-rat Abs are induced following administration of PC61, these Abs do not neutralise circulating PC61 or block the PC61 binding site (data not shown). This failure to enhance tumour rejection may however be attributable to codepletion of activated nonregulatory T cells.

#### Tumour incidence in IFN*γ*^−/−^, PC61-treated mice

**Figure 3:**
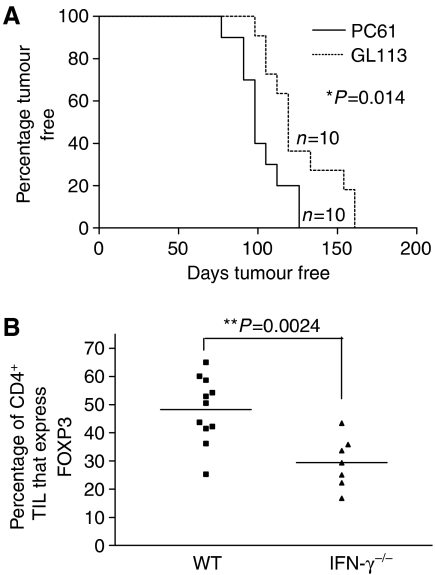
IFN*γ* deficiency abrogates the ability of Treg depletion to reduce tumour development. IFN*γ*^−/−^ and WT mice were treated with 1 mg of either PC61 or isotype control GL113 mAb before the injection of MCA and tumour development was monitored (**A**). Log-rank statistical analysis was used to determine the difference in tumour induction between experimental groups. Single-cell suspensions were prepared from tumours excised from WT and IFN*γ*^−/−^ tumour bearing mice and stained with anti-CD4 FITC, anti-CD25 PerCP-Cy5.5 and anti-FOXP3 PE-specific mAbs. The percentage of CD4^+^ TIL that expressed FOXP3 in IFN*γ*^−/−^ and WT derived tumours was compared and analysed using an unpaired student's *t*-test (**B**).

IFN*γ* plays an important role in controlling development of MCA-induced tumours (Kaplan *et al*, 1998; Shankaran *et al*, 2001). In agreement with these previous studies, we found that whereas only 70% of WT mice had developed tumours by day 200 after injection with 400 *μ*g of MCA, a similar dose induced tumours in all IFN*γ*^−/−^ mice by day 160 (compare Figure 2D to Figure 3A). To gain an insight into the mechanism of tumour rejection in Treg-depleted animals, we determined whether treatment of IFN*γ*^−/−^ mice with CD25-specific mAbs offered protection against the development of MCA-induced tumours. Depletion of Tregs in these mice did not offer any protection against tumour development. In fact, tumours appeared significantly earlier in IFN*γ*^−/−^ mice treated with CD25-specific mAbs compared to mice receiving the control mAbs (Figure 3A). Interestingly, a much smaller percentage (approximately 40% reduction) of Tregs was observed in tumours excised from IFN*γ*^−/−^ mice compared to those from WT mice (Figure 3B). Similarly, no enrichment of Tregs was observed in the TDLN of IFN*γ*^−/−^ mice (data not shown). These data imply that expansion and/or migration of Tregs in response to a developing tumour is impaired in the absence of IFN*γ*.

### Circulating dsDNA specific autoantibodies in Treg-depleted mice

**Figure 4:**
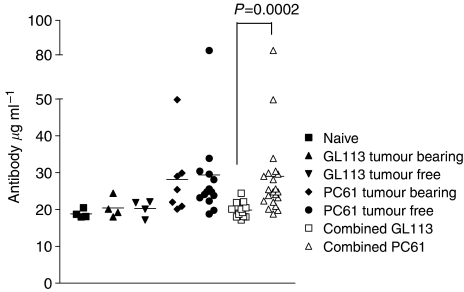
Autoantibody levels are increased in Treg-depleted mice. Serum from naïve mice and mice injected with MCA after treatment with PC61 or GL113 was collected and tested for dsDNA-specific IgM autoantibody and analysed with an unpaired student's *t*-test.

As a reduction in Treg numbers has been associated with the development of autoimmunity, circulating autoantibodies were analysed in mice that remained tumour free following Treg depletion. Mice treated with PC61 had a significantly elevated concentration of circulating dsDNA-specific IgM autoantibodies compared to mice that were not injected with PC61; however, there was no difference in dsDNA-specific autoantibody levels in mice treated with PC61 and that remained tumour free compared to tumour-bearing mice that received PC61 (Figure 4).

## DISCUSSION

The aim of the work described here was to directly investigate whether Tregs impinge on immune surveillance of developing tumours *in vivo*. In this study, we used MCA, a carcinogen that has been widely used to identify a role of a series of immune cell types and signalling molecules that suppress tumour development. The study revealed several interesting findings. We first found a significant enrichment of CD4^+^FOXP3^+^ Tregs in tumours developing *in vivo*, where approximately half of total CD4^+^ TIL were FOXP3^+^. Moreover, injection of mice with the CD25-specific mAb, PC61, resulted in a transient depletion of a proportion of the Tregs before MCA injection and a significantly reduced tumour incidence. We believe that interesting parallels can be drawn between this work and a study performed 20 years previously by Prehn and Kojima (1986). Working on the premise that ‘the presence of autoimmunity implies an increased antitumour immunity’, Prehn and Kojima (1986)found that 3-day thymectomy inhibited development of tumours using a high dose of MCA. Based on the observation that thymic production of CD25^+^ T cells, capable of suppressing the activity of autoreactive T cells, begins only on day 3 after birth in mice (Asano *et al*, 1996). It appears that inhibition of tumour development by day-3 thymectomy and by injection of the CD25-specific mAb, PC61, occurs by ablation of Treg activity. Ambrosino *et al* (2006) recently described treatment of Erbb2 transgenic mice with the CD25-specific mAb, PC61. These animals develop multiple mammary carcinomas as a result of overexpression of the Erbb2 oncogene. PC61-treated mice demonstrated reduced carcinoma multiplicity and a concomitant increase in immune responses to p185, the protein product of Errb2 (Ambrosino *et al*, 2006). In our study, we found that circulating dsDNA-specific IgM Abs were elevated in Treg-depleted, MCA-injected mice compared to control mice implying that Treg depletion drives autoreactivity. Collectively, these studies indicate that Tregs inhibit immune surveillance and support the premise that the involvement of Tregs in this process is a consequence of their role in limiting self-reactive immune responses.

The key question arising from our data relates to the type of effector cells promoting tumour rejection after depletion of Tregs. As Tregs have been shown to suppress both innate and adaptive immune responses to tumours (reviewed by Betts *et al*, 2006), it is likely that multiple arms of the immune system including both antigen-specific T cells and nonspecific inflammatory responses are more effective following Treg depletion. In the case of MCA-induced tumours, Nishikawa *et al* (2005) recently showed that Tregs, induced by immunisation with a SEREX-defined self-antigen expressed in tumour cells, inhibited NK and NKT cells capable of inhibiting the development of MCA-induced tumours, thus indicating that these cells are targets of Treg activity and important antitumour effector cells (Nishikawa *et al*, 2005). Ongoing studies in our laboratory are seeking to define precisely the nature of the antitumour responses induced in Treg-depleted, MCA-injected mice.

Previous studies have highlighted a critical role for IFN*γ* in controlling the development of MCA-induced tumours (Kaplan *et al*, 1998; Shankaran *et al*, 2001). IFN*γ* can control tumour growth directly through proapoptotic, antiangiogenic and antiproliferative effects and indirectly, by facilitating induction of antitumour innate and adaptive immune responses (reviewed by Smyth *et al*, 2006). Our study shows that the effect of depleting Tregs is lost in mice lacking IFN*γ*. This may be because immune responses uncovered by Treg depletion mediate tumour rejection through production of IFN*γ* and/or that immune responses uncovered by Treg depletion cannot compensate for the lack of IFN*γ*, which is critical for tumour control even in the absence of Tregs. Interestingly and in contrast to our observations in wild-type mice, tumours appeared slightly yet significantly earlier in IFN*γ*^−/−^ mice receiving CD25-specific mAbs compared to those that received control mAbs. This finding suggests a fundamental difference in the response of wild-type and IFN*γ*^−/−^ mice to MCA injection. It is possible that CD25-specific mAbs deplete important tumour-induced effector cells in IFN*γ*^−/−^ mice. Alternatively, the impact of Treg activity on tumour development may differ between IFN*γ*^−/−^ and wild-type mice. Tregs have been shown in some experimental models to *suppress* tumour progression by inhibiting inflammatory responses that would otherwise promote tumour development (Erdman *et al*, 2003). Such inflammatory responses might be induced in response to MCA injection in IFN*γ*^−/−^ mice, thus the overall influence of Tregs would be to suppress tumour progression through limiting these responses. We did however observe that IFN*γ*^−/−^ mice, when challenged with MCA, demonstrate a reduction of the percentage of CD4^+^FOXP3^+^ Tregs within tumours and TDLN. As mentioned earlier, this may reflect a role for IFN*γ* in expansion/migration of Treg cells or an overall increase in the expansion of effector T cells in the IFN*γ*^−/−^ animals. Interestingly, the function of Tregs has previously been shown to be impaired in these mice (Kelchtermans *et al*, 2005). Furthermore, Wang *et al* (2006) recently showed that IFN*γ* is important for the conversion of CD4^+^CD25^−^FOXP3^−^ cells to CD4^+^CD25^+^FOXP3^+^ Tregs, thus it is possible that those CD4^+^FOXP3^+^ Tregs in MCA-induced tumours are derived from CD4^+^CD25^−^FOXP3^−^ cells by a process, which is IFN*γ* dependent.

An accumulation of data obtained in studies of patients with cancer does support the concept that Treg depletion will have a beneficial effect in cancer immunotherapy. Overall, the results of this study support this premise by revealing a role for Tregs in suppressing effective immune surveillance of carcinogen-induced tumours in intact animals. A more cautionary note, implied by the findings of this study is that the nature of the ongoing immune response to the tumour may alter the outcome of Treg depletion, in some cases favouring tumour progression rather than tumour control. Further studies are clearly required to clarify this issue.

## References

[bib1] Ambrosino E, Spadaro M, Iezzi M, Curcio C, Forni G, Musiani P, Wei WZ, Cavallo F (2006) Immunosurveillance of Erbb2 carcinogenesis in transgenic mice is concealed by a dominant regulatory T-cell self-tolerance. Cancer Res 66: 7734–7740 16885376 10.1158/0008-5472.CAN-06-1432

[bib2] Asano M, Toda M, Sakaguchi N, Sakaguchi S (1996) Autoimmune disease as a consequence of developmental abnormality of a T cell subpopulation. J Exp Med 184: 387–396 8760792 10.1084/jem.184.2.387 PMC2192701

[bib3] Baecher-Allan C, Brown JA, Freeman GJ, Hafler DA (2001) CD4+CD25high regulatory cells in human peripheral blood. J Immunol 167: 1245–1253 11466340 10.4049/jimmunol.167.3.1245

[bib4] Bennett CL, Christie J, Ramsdell F, Brunkow ME, Ferguson PJ, Whitesell L, Kelly TE, Saulsbury FT, Chance PF, Ochs HD (2001) The immune dysregulation, polyendocrinopathy, enteropathy, X-linked syndrome (IPEX) is caused by mutations of FOXP3. Nat Genet 27: 20–21 11137993 10.1038/83713

[bib5] Betts GJ, Clarke SL, Richards HE, Godkin AJ, Gallimore AM (2006) Regulating the immune response to tumours. Adv Drug Deliv Rev 58: 948–961 17070961 10.1016/j.addr.2006.05.006

[bib6] Brunkow ME, Jeffery EW, Hjerrild KA, Paeper B, Clark LB, Yasayko SA, Wilkinson JE, Galas D, Ziegler SF, Ramsdell F (2001) Disruption of a new forkhead/winged-helix protein, scurfin, results in the fatal lymphoproliferative disorder of the scurfy mouse. Nat Genet 27: 68–73 11138001 10.1038/83784

[bib7] Burnet M (1957) Cancer: a biological approach. III. Viruses associated with neoplastic conditions. IV. practical applications. Br Med J 841–847 13413231 10.1136/bmj.1.5023.841 PMC1973618

[bib8] Clarke SL, Betts GJ, Plant A, Wright KL, El-Shanawany TM, Harrop R, Torkington J, Rees BI, Williams GT, Gallimore AM, Godkin AJ (2006) CD4CD25FOXP3 regulatory T cells suppress anti-tumor immune responses in patients with colorectal cancer. PLoS ONE 1: e129 17205133 10.1371/journal.pone.0000129 PMC1762416

[bib9] Curiel TJ, Coukos G, Zou L, Alvarez X, Cheng P, Mottram P, Evdemon-Hogan M, Conejo-Garcia JR, Zhang L, Burow M, Zhu Y, Wei S, Kryczek I, Daniel B, Gordon A, Myers L, Lackner A, Disis ML, Knutson KL, Chen L, Zou W (2004) Specific recruitment of regulatory T cells in ovarian carcinoma fosters immune privilege and predicts reduced survival. Nat Med 10: 942–949 15322536 10.1038/nm1093

[bib10] Erdman SE, Poutahidis T, Tomczak M, Rogers AB, Cormier K, Plank B, Horwitz BH, Fox JG (2003) CD4^+^ CD25^+^ regulatory T lymphocytes inhibit microbially induced colon cancer in Rag2-deficient mice. Am J Pathol 162: 691–702 12547727 10.1016/S0002-9440(10)63863-1 PMC1851156

[bib11] Fontenot JD, Rasmussen JP, Williams LM, Dooley JL, Farr AG, Rudensky AY (2005) Regulatory T cell lineage specification by the forkhead transcription factor foxp3. Immunity 22: 329–341 15780990 10.1016/j.immuni.2005.01.016

[bib12] Ghiringhelli F, Puig PE, Roux S, Parcellier A, Schmitt E, Solary E, Kroemer G, Martin F, Chauffert B, Zitvogel L (2005) Tumor cells convert immature myeloid dendritic cells into TGF-beta-secreting cells inducing CD4^+^CD25^+^ regulatory T cell proliferation. J Exp Med 202: 919–929 16186184 10.1084/jem.20050463 PMC2213166

[bib13] Golgher D, Jones E, Powrie F, Elliott T, Gallimore A (2002) Depletion of CD25^+^ regulatory cells uncovers immune responses to shared murine tumor rejection antigens. Eur J Immunol 32: 3267–3275 12555672 10.1002/1521-4141(200211)32:11<3267::AID-IMMU3267>3.0.CO;2-1

[bib14] Kaplan DH, Shankaran V, Dighe AS, Stockert E, Aguet M, Old LJ, Schreiber RD (1998) Demonstration of an interferon gamma-dependent tumor surveillance system in immunocompetent mice. Proc Natl Acad Sci USA 95: 7556–7561 9636188 10.1073/pnas.95.13.7556 PMC22681

[bib15] Kelchtermans H, De Klerck B, Mitera T, Van Balen M, Bullens D, Billiau A, Leclercq G, Matthys P (2005) Defective CD4^+^CD25^+^ regulatory T cell functioning in collagen-induced arthritis: an important factor in pathogenesis, counter-regulated by endogenous IFN-gamma. Arthritis Res Ther 7: R402–R415 15743488 10.1186/ar1500 PMC1065335

[bib16] Lowenthal JW, Corthesy P, Tougne C, Lees R, MacDonald HR, Nabholz M (1985) High and low affinity IL 2 receptors: analysis by IL 2 dissociation rate and reactivity with monoclonal anti-receptor antibody PC61. J Immunol 135: 3988–3994 3934270

[bib17] Nishikawa H, Kato T, Tawara I, Takemitsu T, Saito K, Wang L, Ikarashi Y, Wakasugi H, Nakayama T, Taniguchi M, Kuribayashi K, Old LJ, Shiku H (2005) Accelerated chemically induced tumor development mediated by CD4^+^CD25^+^ regulatory T cells in wild-type hosts. Proc Natl Acad Sci USA 102: 9253–9257 15961541 10.1073/pnas.0503852102 PMC1166632

[bib18] Onizuka S, Tawara I, Shimizu J, Sakaguchi S, Fujita T, Nakayama E (1999) Tumor rejection by *in vivo* administration of anti-CD25 (interleukin-2 receptor alpha) monoclonal antibody. Cancer Res 59: 3128–3133 10397255

[bib19] Prehn LM, Kojima A (1986) Paradoxical effect of three-day thymectomy on sarcogenesis in the mouse with different dosages of methylcholanthrene. Cancer Res 46: 4971–4972 3756859

[bib20] Roncador G, Brown PG, Maestre L, Hue S, Martinez-Torrecuadrada JL, Ling KL, Pratap S, Toms C, Fox BC, Cerundolo V, Powrie F, Banham AH (2005) Analysis of FOXP3 protein expression in human CD4+CD25+regulatory T cells at the single-cell level. Eur J Immunol 35: 1681–1691 15902688 10.1002/eji.200526189

[bib21] Sakaguchi S (2005) Naturally arising Foxp3-expressing CD25^+^CD4^+^ regulatory T cells in immunological tolerance to self and non-self. Nat Immunol 6: 345–352 15785760 10.1038/ni1178

[bib22] Shankaran V, Ikeda H, Bruce AT, White JM, Swanson PE, Old LJ, Schreiber RD (2001) IFNgamma and lymphocytes prevent primary tumour development and shape tumour immunogenicity. Nature 410: 1107–1111 11323675 10.1038/35074122

[bib23] Shimizu J, Yamazaki S, Sakaguchi S (1999) Induction of tumor immunity by removing CD25^+^CD4^+^ T cells: a common basis between tumor immunity and autoimmunity. J Immunol 163: 5211–5218 10553041

[bib24] Smyth MJ, Dunn GP, Schreiber RD (2006) Cancer immunosurveillance and immunoediting: the roles of immunity in suppressing tumor development and shaping tumor immunogenicity. Adv Immunol 90: 1–50 16730260 10.1016/S0065-2776(06)90001-7

[bib25] Sutmuller RP, van Duivenvoorde LM, van Elsas A, Schumacher TN, Wildenberg ME, Allison JP, Toes RE, Offringa R, Melief CJ (2001) Synergism of cytotoxic T lymphocyte-associated antigen 4 blockade and depletion of CD25(+) regulatory T cells in antitumor therapy reveals alternative pathways for suppression of autoreactive cytotoxic T lymphocyte responses. J Exp Med 194: 823–832 11560997 10.1084/jem.194.6.823 PMC2195955

[bib26] Wang Z, Hong J, Sun W, Xu G, Li N, Chen X, Liu A, Xu L, Sun B, Zhang JZ (2006) Role of IFN-gamma in induction of Foxp3 and conversion of CD4+ CD25− T cells to CD4+ Tregs. J Clin Invest 116: 2434–2441 16906223 10.1172/JCI25826 PMC1533873

[bib27] Wolf D, Wolf AM, Rumpold H, Fiegl H, Zeimet AG, Muller-Holzner E, Deibl M, Gastl G, Gunsilius E, Marth C (2005) The expression of the regulatory T cell-specific forkhead box transcription factor FoxP3 is associated with poor prognosis in ovarian cancer. Clin Cancer Res 11: 8326–8331 16322292 10.1158/1078-0432.CCR-05-1244

